# One-year pilot study on the effects of nitisinone on melanin in patients with OCA-1B

**DOI:** 10.1172/jci.insight.124387

**Published:** 2019-01-24

**Authors:** David R. Adams, Supriya Menezes, Ramon Jauregui, Zaheer M. Valivullah, Bradley Power, Maria Abraham, Brett G. Jeffrey, Angel Garced, Ramakrishna P. Alur, Denise Cunningham, Edythe Wiggs, Melissa A. Merideth, Pei-Wen Chiang, Shanna Bernstein, Shosuke Ito, Kazumasa Wakamatsu, Rhona M. Jack, Wendy J. Introne, William A. Gahl, Brian P. Brooks

**Affiliations:** 1 National Human Genome Research Institute, NIH, Department of Health and Human Services, Bethesda, Maryland, USA.; 2 Emmes, Rockville, Maryland, USA.; 3 National Eye Institute and; 4 National Institute of Neurological Disease and Stroke, NIH, Department of Health and Human Services, Bethesda, Maryland, USA.; 5 Molecular Vision Laboratory, Hillsboro, Oregon, USA.; 6 Nutrition Department, NIH Clinical Center, Bethesda, Maryland, USA.; 7 Department of Chemistry, Fujita Health University School of Health Sciences, Toyoake, Aichi, Japan.; 8 Seattle Children’s Hospital, Department of Pathology & Laboratory Medicine, Seattle, Washington, USA.

**Keywords:** Genetics, Ophthalmology, Genetic diseases

## Abstract

**BACKGROUND.** Oculocutaneous albinism (OCA) results in reduced melanin synthesis, skin hypopigmentation, increased risk of UV-induced malignancy, and developmental eye abnormalities affecting vision. No treatments exist. We have shown that oral nitisinone increases ocular and fur pigmentation in a mouse model of one form of albinism, OCA-1B, due to hypomorphic mutations in the *Tyrosinase* gene.

**METHODS.** In this open-label pilot study, 5 adult patients with OCA-1B established baseline measurements of iris, skin, and hair pigmentation and were treated over 12 months with 2 mg/d oral nitisinone. Changes in pigmentation and visual function were evaluated at 3-month intervals.

**RESULTS.** The mean change in iris transillumination, a marker of melanin, from baseline was 1.0 ± 1.54 points, representing no change. The method of iris transillumination grading showed a high intergrader reliability (intraclass correlation coefficient ≥ 0.88 at each visit). The number of letters read (visual acuity) improved significantly at month 12 for both eyes (right eye, OD, mean 4.2 [95% CI, 0.3, 8.1], *P* = 0.04) and left eye (OS, 5 [1.0, 9.1], *P* = 0.003). Skin pigmentation on the inner bicep increased (M index increase = 1.72 [0.03, 3.41], *P* = 0.047). Finally, hair pigmentation increased by both reflectometry (M index [17.3 {4.4, 30.2}, *P* = 0.01]) and biochemically.

**CONCLUSION.** Nitisinone did not result in an increase in iris melanin content but may increase hair and skin pigmentation in patients with OCA-1B. The iris transillumination grading scale used in this study proved robust, with potential for use in future clinical trials.

**TRIAL REGISTRATION. **ClinicalTrials.gov NCT01838655.

**FUNDING.** Intramural program of the National Eye Institute.

## Abstract 2

Oral nitisinone may improve melanin pigmentation in patients with the OCA-1B form of albinism due to hypomorphic mutations in the tyrosinase gene.

## Introduction

Oculocutaneous albinism (OCA) is a genetically heterogeneous, autosomal recessive condition characterized by absent or reduced melanin pigment in the eyes, hair, and skin (1–3). Melanins are complex biomolecules made in melanocytes and the retinal pigment epithelium (RPE) and stored in subcellular organelles called melanosomes. They can be divided into two broad biochemical categories: eumelanin (brown-black melanin) and pheomelanin (red-brown melanin, which contains sulfur atoms). To date, biallelic mutations in 6 distinct genes (7 loci, *OCA1–OCA7*) have been described in nonsyndromic OCA (4); of these, mutations in the tyrosinase (*TYR*, *OCA1*) gene and the *OCA2* gene (formerly, the *P gene*) are the most prevalent forms of OCA in North America (3, 5). Most, if not all, forms of human albinism are the direct or indirect result of reduced tyrosinase activity in the melanosome. Indirect causes include decreasing the stability of the tyrosinase protein (leading to its degradation) and altering the ionic composition/pH of the melanosome (6–8).

Melanin plays important roles in the health of the skin and the eyes. In the skin, melanin acts as a broadband protectant against UV light and has free radical scavenging and antioxidant properties (9). As such, patients with albinism are thought to have an increased risk of skin cancers, such as basal cell carcinoma, squamous cell carcinoma, and melanoma (10, 11). Interestingly, de Vijlder and colleagues have proposed that the presence of relatively normal levels of pheomelanin, in combination with significantly decreased levels of eumelanin, as seen in OCA2, may put such people at greater risk of skin cancer than observed in the complete absence of all forms of melanin (OCA1A) (12).

Furthermore, patients with albinism experience a decrease in best-corrected visual acuity and stereopsis due to developmental eye abnormalities, namely hypoplasia of the fovea (a highly specialized, avascular area of the neural retina with dense cone photoreceptor packing) and abnormal decussation of retinal ganglion cell axons at the level of the optic chiasm (13–17). Visual difficulties may be exacerbated by nystagmus (with or without anomalous head posture for a null point), high refractive errors, strabismus, and amblyopia (15, 18). In addition, the lack of melanin pigment in the iris (demonstrated by iris transillumination [TI] on exam) and the fundus are thought to contribute to the photoaversion experienced by many albinism patients. Current treatments for vision-related morbidity in albinism are limited to supportive measures, such as correction of refractive error, sun protection, and low vision aids.

The precise reason why a defect in melanin production in one tissue type (i.e., the RPE) leads to developmental abnormalities in an adjacent tissue (the neural retina) is unknown. Although foveal differentiation begins around 22 weeks of gestation, it continues for months postnatally, offering a potential therapeutic window (19, 20). Regardless of the precise mechanism, we hypothesized that if we were able to improve melanin pigmentation in the posterior segment of patients with albinism during this critical window of foveal development that we would have a positive effect on future best-corrected visual acuity. Even treatment in older children and adults might improve skin and hair melanin content, reducing the risk of UV-induced skin cancer. An increase in iris and/or fundus pigmentation could also limit the amount of stray light reaching the neurosensory retina and, therefore, light aversion.

Recently, our group reported that treatment with oral nitisinone in a mouse model of one form of albinism, OCA-1B, due to hypomorphic mutations in the *Tyr* gene, improved the fur and ocular melanin (21). Nitisinone is an FDA-approved medication used clinically in the treatment of tyrosinemia, type 1 (hepatorenal tyrosinemia [HT-1]) caused by deficient fumarylacetoacetate hydrolase (FAH) activity (reviewed in ref. 22). By inhibiting an enzyme upstream in the tyrosine catabolism pathway (4-hydroxyphenylpyruvate dioxygenase), nitisinone prevents the accumulation of toxic byproducts of FAH deficiency (e.g., succinylacetone). As a “side effect,” nitisinone treatment further raises the plasma concentration of tyrosine in HT-1 patients. We have demonstrated that small doses of oral nitisinone in OCA-1B mice fed ad libitum similarly increases plasma tyrosine and increases melanin pigmentation in the fur and eyes (21). In vitro modeling and enzymology data suggest that a potential mechanism of the observed increase in melanin is due to stabilization of the mutant tyrosinase protein with saturating levels of tyrosine, its substrate.

We therefore hypothesized that oral nitisinone would similarly increase ocular, skin, and/or hair pigmentation in humans with OCA-1B. In this pilot study, adults were chosen (rather than children) to (a) demonstrate drug safety in an albinism population and (b) to establish proof-of-concept findings that potentially could be applied in younger populations, in whom a greater effect on vision development might be anticipated. Melanin pigmentation, rather than visual acuity, was the primary focus, because the visual system of adults is largely mature; we would not expect to see an increase in best-corrected visual acuity. We evaluate the effect of a fixed, daily, low dose of oral nitisinone on 5 adult patients with molecularly confirmed OCA-1B over the course of 1 year. A similar dose in a trial of adult patients with alkaptonuria resulted in minimal side effects, 95% inhibition of the 4-hydroxyphenylpyruvate dioxygenase enzyme, and a 10-fold elevation of plasma tyrosine concentrations (23). We explore the utility of a novel iris TI grading scale as our primary clinical outcome measure for this and other, future clinical trials (24). Secondary outcome measures included changes in melanin pigmentation in the skin, hair, and other parts of the eye as well as visual acuity. Although we observe no favorable significant change in our primary outcome measure (iris TI), the reliability and clinical utility of the scale was high, favoring its use in future trials. Visual acuity improved overall in a statistically significant, but not clinically significant, fashion. Importantly, we also observe a small, but significant, increase in hair and skin melanin, signaling a potential nonocular therapeutic effect. This study may serve as a basis for testing nitisinone and other pharmacologic agents in future clinical trials.

## Results

### Subject characteristics.

Five participants (3 females, 2 males) enrolled and completed the 1-year treatment trial. Table 1 presents the demographic and baseline characteristics. Age ranged from 24 years to 52 years. Visual acuity at enrollment ranged from 20/40 (0.30 LogMAR) to 20/125 (0.80 LogMAR) in the better-seeing eye. Four subjects had at least 2 likely pathogenic or pathogenic mutations in *TYR*; subject 4 had 1 pathogenic variant in *TYR* (p.T373L) and 1 pathogenic variant in *TYRP1* (*OCA3*, p.R93H). All 5 participants responded to daily oral nitisinone with an approximately 10-fold increase in steady-state plasma tyrosine concentrations (Supplemental Figure 1; supplemental material available online with this article; https://doi.org/10.1172/jci.insight.124387DS1). These values are several-fold above the experimental *K_m_* for tyrosine (0.16 ± 0.04 mM), determined using purified recombinant human tyrosinase (25). Plasma tyrosine concentrations remained stable while on nitisinone and normalized to baseline when medication was discontinued.

### Change in iris and fundus pigmentation.

**Figure 1:**
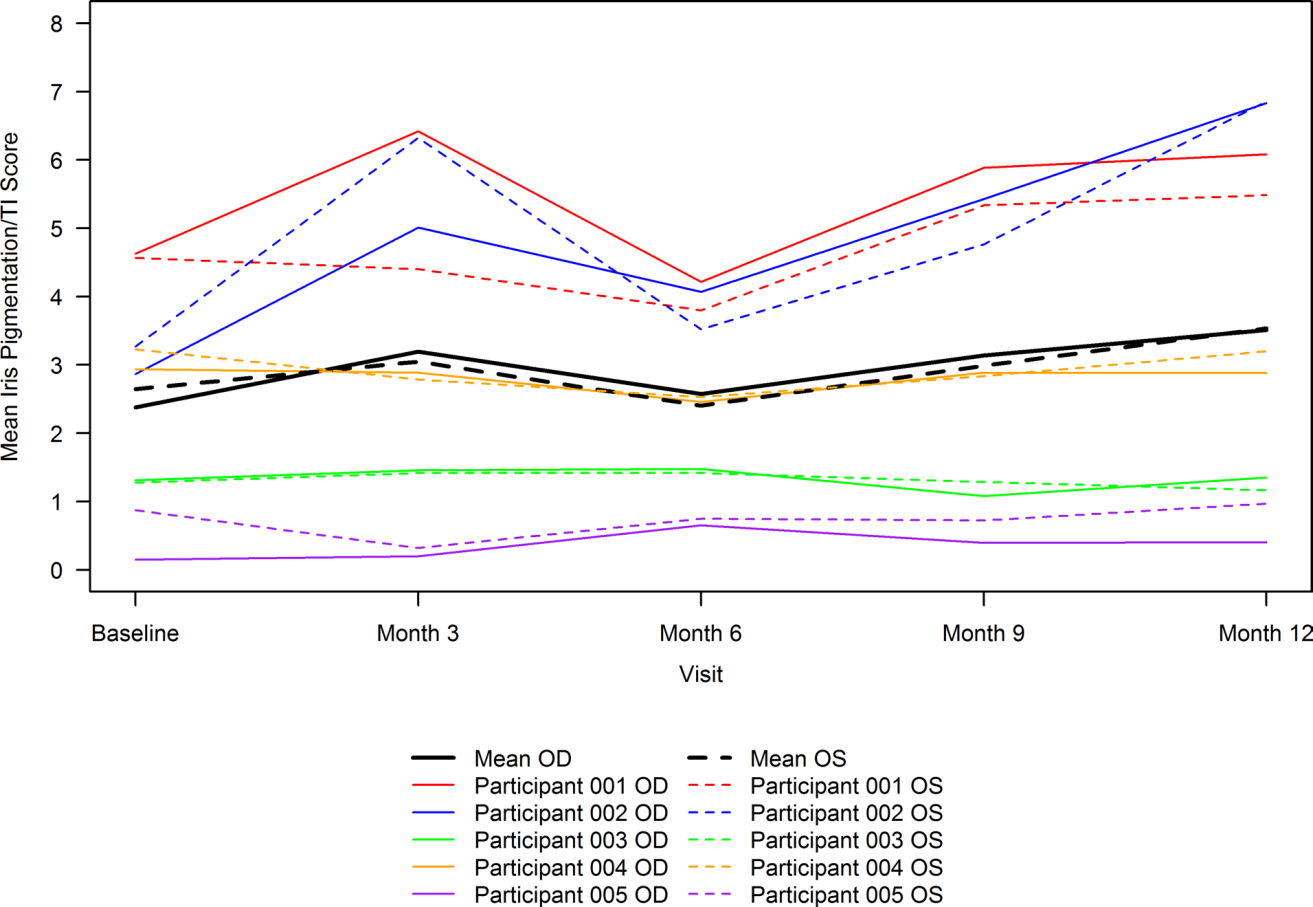
Mean 8-point iris pigmentation/transillumination score over time by participant eye and overall. Mean 8-point iris pigmentation/transillumination (TI) scores were calculated as the mean across all 18 graders for the 2 images of each eye for a participant at each visit. The solid lines correspond to oculus dexter (OD), and dashed lines correspond to oculus sinister (OS) measurements. The colored lines correspond to individual participant measurements, and the black lines correspond to mean of the scores (*n* = 5) across all participants for each eye. An overall increase in score, implying a decrease in melanin content, was noted when each eye was assessed separately (mean [*n* = 5] change from baseline at 12 months of OD 1.1 [1.70] and OS 0.9 [1.56] points) and together (data not shown) (mean [*n* = 10] change from baseline at 12 months of oculus uterque/OU 1.0 [1.54] points; *P* = 0.01 based on a longitudinal model fit using data through month 12).

To assess whether nitisinone improved melanin pigmentation, we used a measure of iris TI through month 12 (24). Note that with this scale, an increased score (increased TI) implies *less* melanin content blocking the light reflected back off the retina. Using this scale, the primary endpoint of mean (SD) of absolute (i.e., regardless of direction of change) mean change from baseline at 12 months was oculus dexter (OD) 1.2 (1.68) and oculus sinister (OS) 0.9 (1.52) points. The mean directional change from baseline at 12 months was OD 1.1 (1.70), OS 0.9 (1.56), and oculus uterque (OU) 1.0 (1.54) points (Table 2), implying an overall decrease in melanin content when each eye was assessed separately and together. The decrease in melanin content when both eyes were assessed together was significant (*P* = 0.01), based on a longitudinal model fit using data through month 12. Looking at each participant individually (Figure 1), participant 002 experienced the maximum change from baseline with a *decrease* in iris melanin, followed by participant 001, while the remaining 3 participants experienced minimal to no change in pigmentation in both eyes from baseline.

The mean across the 18 graders of the average grade provided by each grader for the 2 images for each participant’s eye at each visit were used in the analysis. A fairly high intergrader reliability was observed, with an intraclass correlation coefficient (ICC) of at least 0.88 at each visit (Table 3). An ICC estimate of 1 indicates perfect agreement and 0 indicates only random agreement, while negative ICC estimates indicate systematic disagreement.

**Figure 2:**
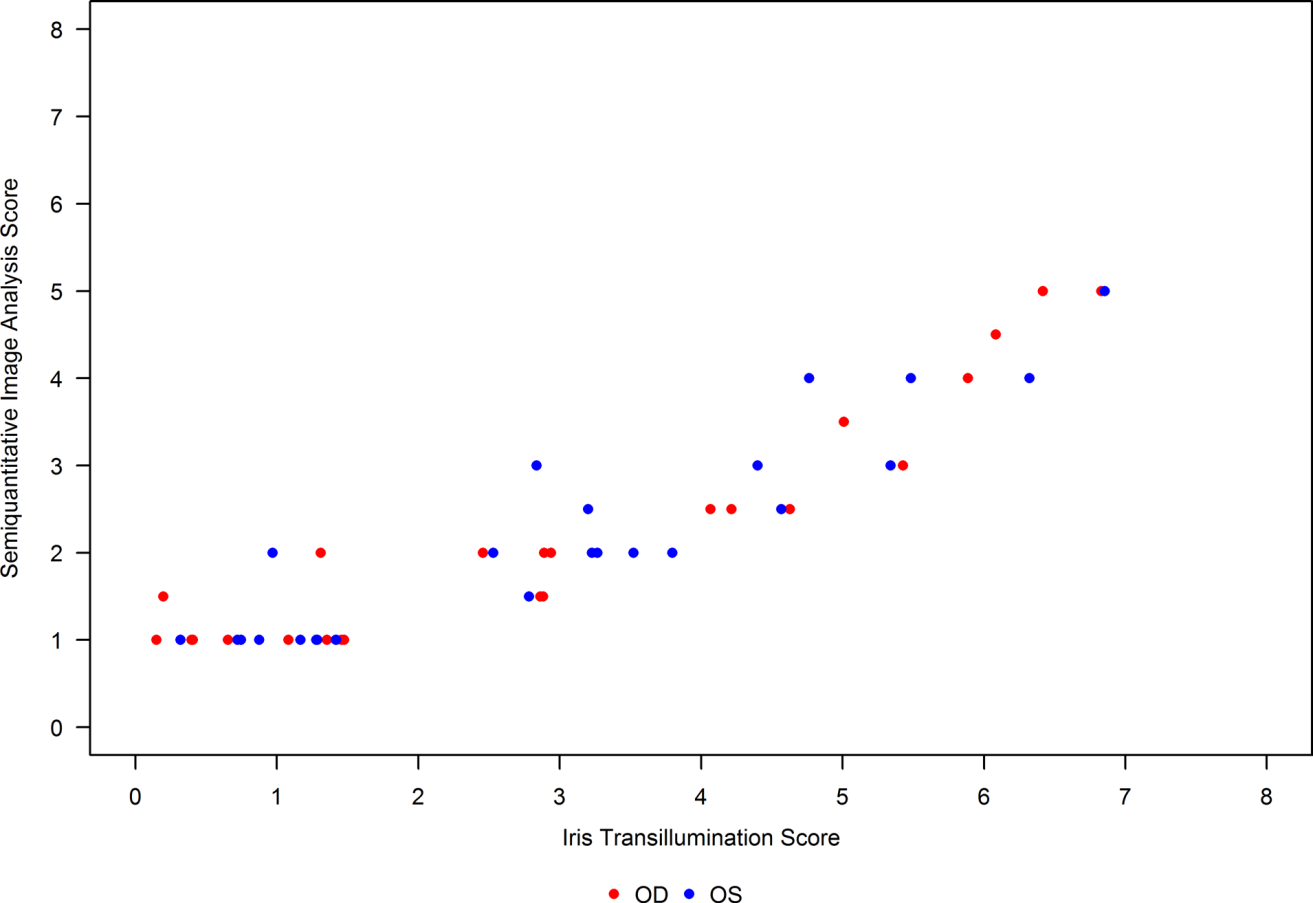
Correlation between mean 8-point iris pigmentation/transillumination and semiquantitative image analysis scores. Mean 8-point iris pigmentation/transillumination (TI)scores were calculated as the mean across all 18 graders for the 2 images of each eye for a participant at each visit. Semiquantitative image analysis scores were calculated as the mean of scores across the 2 images of each eye for a participant at each visit. Oculus dexter (OD) and oculus sinister (OS) values of mean 8-point iris pigmentation/TI and semiquantitative image analysis scores are plotted. These scores were found to be highly correlated with a Spearman’s rank correlation coefficient of 0.9, although the range of semiquantitative image analysis scores assigned was lower than that of mean 8-point iris pigmentation/TI scores.

Semiquantitative iris TI image analysis supplemented the results observed from iris pigmentation/TI scores. On this scale too, an increased score implied *less* melanin content. The mean (SD) of mean change from baseline at 12 months was OD 0.8 (1.89), OS 1.2 (1.15), and OU 1.0 (1.49) (Table 4), corresponding to a decrease in iris melanin. These scores were highly correlated with the iris pigmentation/TI scores, with a Spearman’s rank correlation coefficient of 0.9 (Figure 2), although the range of semiquantitative image analysis scores assigned was lower than that of iris pigmentation/TI scores.

A qualitative assessment of change in fundus pigmentation as judged by fundus photographs did not reveal any change in either direction at any of the visits.

### Change in visual acuity.

**Figure 3:**
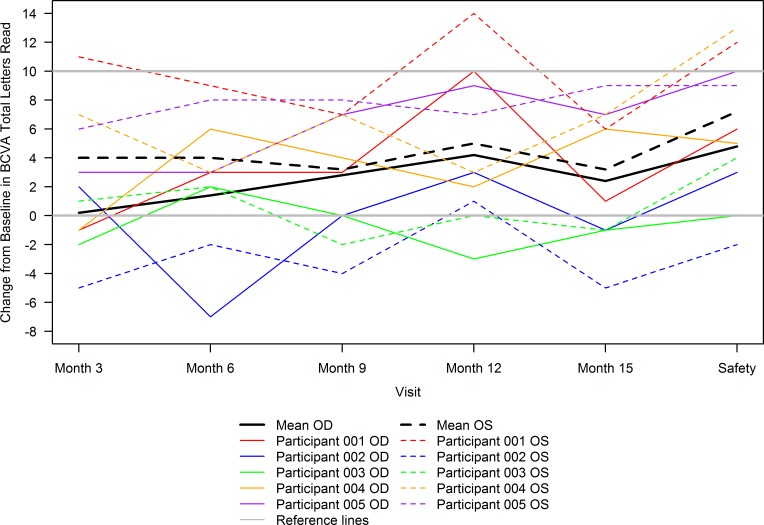
Change from baseline in best-corrected visual acuity total letters read over time by participant eye and overall. Gray solid lines are reference lines drawn at no change and 10-letter increase in best-corrected visual acuity (BCVA) total letters read from baseline. All other solid lines correspond to oculus dexter (OD), and dashed lines correspond to oculus sinister (OS) measurements. The colored lines correspond to individual participant measurements, and the black lines correspond to mean (*n* = 5) across all participants for each eye. In general, an increase in total letters read was seen at all follow-up visits from baseline. Longitudinal models fit using data through safety visit revealed that total letters read significantly improved from baseline at month 12 for OD (mean [95% CI] change from baseline of 4.2 [0.3, 8.1], *P* = 0.04) and OS (mean [95% CI] change from baseline of 5.0 [1.0, 9.1], *P* = 0.02) and, subsequently, at safety follow-up visit for OD (mean [95% CI] change from baseline of 4.8 [0.64, 8.96], *P* = 0.03) and OS (mean [95% CI] change from baseline of 7.2 [2.79, 11.61], *P* = 0.003).

Overall, an increase in total letters read was seen at all follow-up visits from baseline (Table 5 and Figure 3). Longitudinal models fit using data through safety visit revealed that total letters read significantly improved from baseline at month 12 for OD (mean [95% CI] change from baseline of 4.2 [0.3, 8.1], *P* = 0.04) and OS (mean [95% CI] change from baseline of 5.0 [1.0, 9.1], *P* = 0.02) and, subsequently, at safety follow-up visit for OD (mean [95% CI] change from baseline of 4.8 [0.64, 8.96], *P* = 0.03) and OS (mean [95% CI] change from baseline of 7.2 [2.79, 11.61], *P* = 0.003). Considering both eyes together, 4.6 letters (95% CI [2.0, 7.2],*P* = 0.001) were gained at month 12.

### Change in ERG and contrast sensitivity.

A consistent trend of improvement or worsening was not observed in contrast sensitivity or retina function, as assessed by ERG. Longitudinal models fit for the maximal response (DA3) amplitude using data from baseline, month 6, and month 12 revealed no change from baseline in this parameter at month 12 in either eye.

### Change in hair pigmentation.

**Figure 4:**
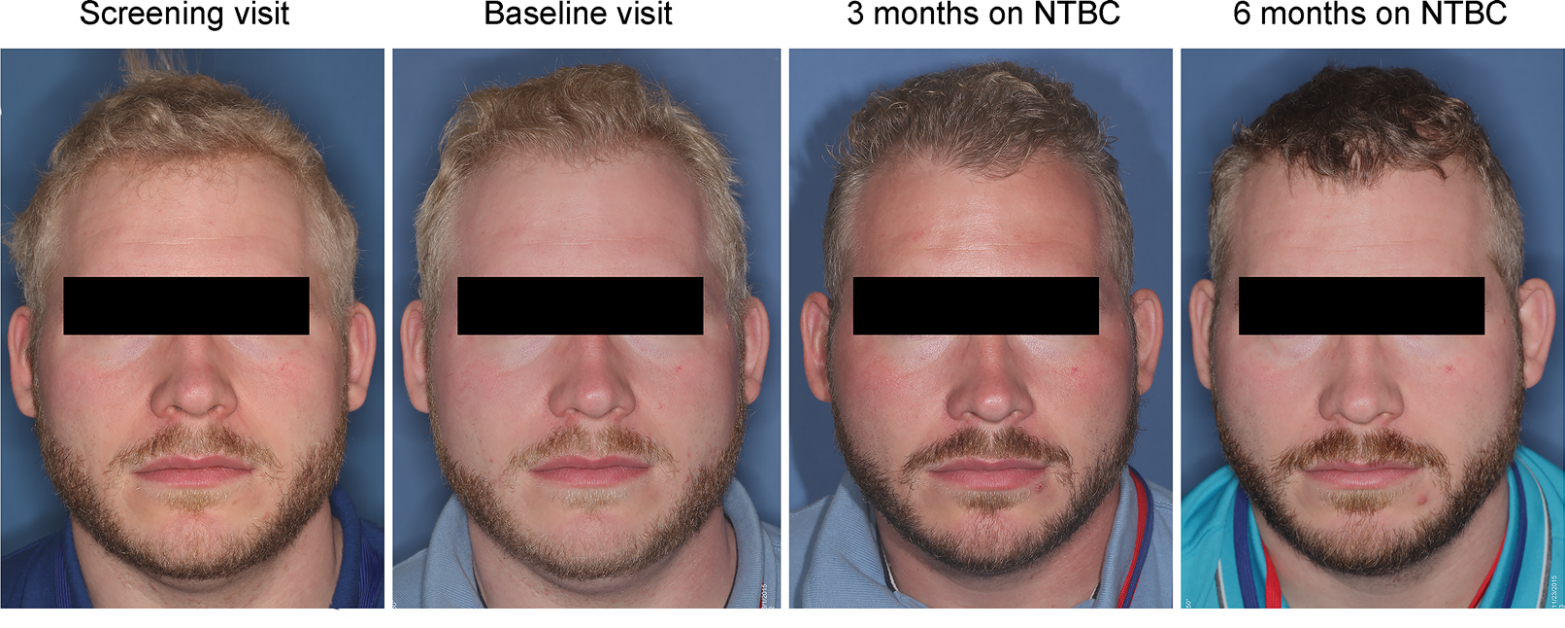
Subject 003 exhibited the greatest visible change in hair and skin pigmentation. He reports that he was previously unable to tan but did so by at 3 months, a summer month. Hair and beard darkening remained at 6 months and until the end of the study. The subject provided written consent for having identifiable images published.

Longitudinal models fit using skin reflectometry data for hair through safety visit revealed a significant increase in hair pigmentation, as assessed by the melanin (M) index (mean [95% CI] increase from baseline of 17.3 [4.4, 30.2], *P* = 0.01) and AM index (mean [95% CI] decrease from baseline of –28.4 [–51.1, –5.7], *P* = 0.02). Four of the five participants (80%) reported qualitative increase in hair pigmentation starting at month 3. In subject 003, the change was clearly notable (Figure 4) in both his hair and beard. He also noted that his ability to tan — previously limited — was improved (see skin reflectometry below).

**Figure 5:**
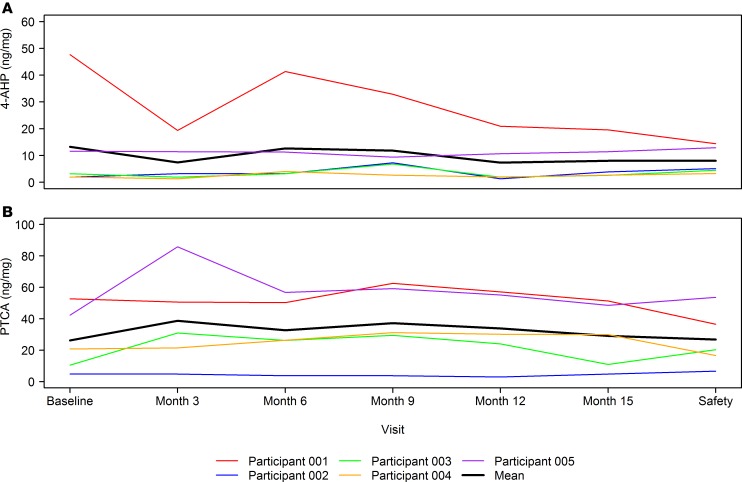
Hair melanin content over time by participant and overall. (**A**) 4-amino-3-hydroxyphenylalanine (4-AHP), a marker of pheomelanin, over time. The colored lines correspond to individual participant measurements, and the black line corresponds to mean (*n* = 5) across all participants. A decrease in 4-AHP was noted at both month 12 (mean [SD] of change from baseline of –5.92 [11.68] ng/mg) and the safety follow-up visit (mean [SD] of change from baseline of –5.24 [15.71] ng/mg). (**B**) Pyrrole-2,3,5-tricarboxylic acid (PTCA), a marker of eumelanin, over time. The colored lines correspond to individual participant measurements, and the black line corresponds to mean (*n* = 5) across all participants. An increase in PTCA was noted at month 12 (mean [SD] of change from baseline of 7.62 [6.43] ng/mg), with almost no change from baseline at the safety follow-up visit (mean [SD] of change from baseline of 0.52 [11.25] ng/mg).

The mean (SD) at baseline of pyrrole-2,3,5-tricarboxylic acid (PTCA) and 4-amino-3-hydroxyphenylalanine (4-AHP), markers of hair melanin content, was 26.22 (20.59) and 13.28 (19.66) ng/mg, respectively. An increase in PTCA was noted at the end of treatment (mean [SD] of change from baseline at month 12 of 7.62 [6.43] ng/mg), with almost no change from baseline at the safety follow-up visit (mean [SD] of change from baseline of 0.52 [11.25] ng/mg). A decrease in 4-AHP was noted at both month 12 (mean [SD] of change from baseline of –5.92 [11.68] ng/mg) and safety follow-up visit (mean [SD] of change from baseline of –5.24 [15.71] ng/mg). Figure 5 shows these parameters over time by participant and overall.

### Change in skin pigmentation.

Longitudinal models fit using skin reflectometry data through the safety visit revealed a significant increase in pigmentation on the inner bicep (mean [95% CI] change from baseline in M index of 1.72 [0.03, 3.41], *P* = 0.047) and a moderately significant increase in pigmentation on the outer forearm (mean [95% CI] change from baseline in M index of 3.4 [–0.5, 7.2], *P* = 0.08) at month 12 compared with baseline. While the former site mostly represents constitutive melanin content at a time point, the latter likely includes contributions of both constitutive and neomelanin (tanning). In the evaluation of M index for inner bicep, a model that fit both random intercept and random slope did not converge (i.e., the numerical algorithm for estimation of the model failed to converge); therefore, a simpler model with only random intercept as a random effect was fit.

A qualitative assessment of change in skin pigmentation revealed that 1 (of 5, 20%) participant had an increase in skin pigmentation starting at month 3 (Figure 4).

### Plasma nitisinone levels.

**Figure 6:**
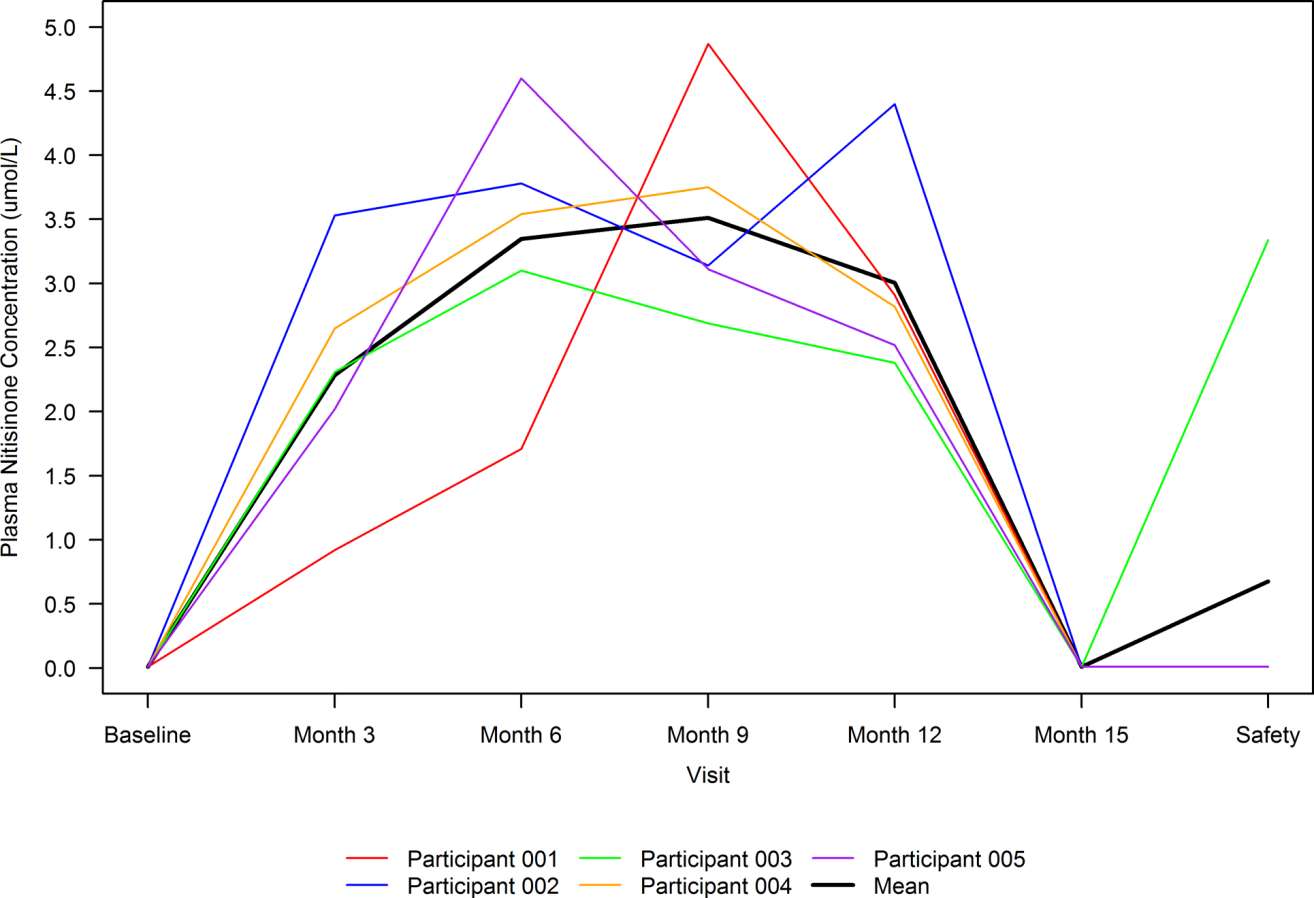
Plasma nitisinone concentration over time by participant and overall. The colored lines correspond to individual participant measurements, and the black line corresponds to mean (*n* = 5) across all participants. Plasma concentrations of nitisinone peaked between months 6 and 12 for participants and dropped to almost nil at month 15, which is 3 months after investigational product (IP) discontinuation. One participant chose the option to restart IP at month 18, before discontinuing it approximately 1.5 months later and completing their safety follow-up visit, resulting in the spike in mean plasma nitisinone concentration at the safety follow-up visit.

Plasma concentrations of nitisinone peaked between months 6 and 12 for participants and dropped to almost nil at month 15, which is 3 months after investigational product (IP) discontinuation (Figure 6). The spike in plasma concentration seen at the safety visit is because IP was restarted at month 18 by 1 of the participants (who chose the option to restart IP at month 18 in the protocol), before it was discontinued approximately 1.5 months later and the safety follow-up visit was completed.

### Safety assessments.

No safety signals were observed in the study. No serious adverse events were reported. A complete list of treatment-emergent adverse events is given in Supplemental Table 2. Specifically, no corneal crystalline deposits — a known complication of hypertyrosinemia — were observed.

Mean intraocular pressure (IOP) for both eyes was roughly constant throughout the follow-up period (data not shown). Assessment of IOP was added after the study was underway; therefore, none of the participants had baseline IOP measurements and the first 2 enrolled participants did not have the month 3 IOP measurements.

A slight increase (improvement) in VFQ-39 total and subscale scores was observed at month 12, except in social functioning and driving subscale scores (Supplemental Table 3). All participants had a score of 100 at baseline for color vision subscale, with no change at month 12. Maximum improvement was observed in peripheral vision (mean [SD] increase from baseline of 20.0 [11.18]), general vision (mean [SD] increase from baseline of 15.0 [21.21]), and ocular pain (mean [SD] increase from baseline of 15.0 [16.30]) subscale scores.

All participants had normal neuropsychological evaluation results, except for 1 participant who had abnormal results at month 12 because CALCAP reaction time was in the impaired range (sequential reaction time *Z*-score of –3.1 and choice reaction time *Z*-score of –8.4). The subject, who was in postgraduate training, had not noticed any change in her reaction time or ability to concentrate during the study. The CALCAP reaction time returned to baseline on retesting 3 months later.

## Discussion

Pilot clinical trials may serve many purposes, all with the aim of informing the design of a larger, definitive clinical trial (26). They may help to evaluate the practicality and/or robustness of a novel outcome variable; explore techniques in data acquisition and analysis; and determine the feasibility of recruitment, retention, and/or randomization. Such an approach seems particularly appropriate in instances such as albinism, where there is no standard treatment, where there is limited experience with conducting trials, and where traditional outcome variables, such as visual acuity, are unlikely to be informative. The purpose of this and other pilot trials is explicitly *not* to formally evaluate the safety, effectiveness, or efficacy of an intervention. Nonetheless, performing analyses on data collected in such a trial may suggest a treatment effect, informing the larger, more definitive follow-on study’s size (power) and scope.

In this pilot clinical trial of oral nitisinone in OCA-1B, we accomplished the above-mentioned goals. We determined that iris TI, although not significantly altered in these subjects, can be robustly measured by graders with only minimal instruction. We observed small, statistically significant improvement in visual acuity that did not rise to the accepted level of clinical significance (i.e., 2 lines of acuity gain/loss on a standard Early Treatment Diabetic Retinopathy Study [ETDRS] chart). Finally, skin reflectometry and hair eumelanin/pheomelanin measurements suggested an increase in melanin synthesis, at least in some subjects. Importantly, this medication was well-tolerated and no serious adverse events occurred. Given the lack of viable alternative medical treatments for albinism, these observations give us reason to pursue the use of nitisinone in younger subjects with albinism, in whom we hypothesize, a larger therapeutic potential.

A significant accomplishment of this study was the evaluation of the robustness of iris TI grading. Iris TI was chosen as the primary outcome variable for several reasons: (a) it was a visible effect in our preclinical model of nitisinone treatment in OCA-1B (21); (b) it is readily photographed in a standardized fashion; and (c) it likely reflects a lack of melanin in the posterior pigment epithelium of the iris, which is contiguous with the RPE of the posterior pole. Because RPE pigmentation cannot be directly observed — most of the melanin pigment observed in fundus photos is from melanocytes in the choroid (27) — we posit that iris TI is a reasonable surrogate for RPE pigmentation. To our knowledge, this study represents the first clinical trial in which iris TI has been used as an outcome variable. Although our measurements suggest a trend of an increase in iris TI, the effect was small, with CIs larger than the observed effect. A qualitative, retrospective review of the primary data suggests that some of the observed variability may be improved with more stringent quality control at the time of image acquisition. Importantly, the intergrader agreement on iris TI scores was quite good (ICC of at least 0.88 for each visit), indicating that this metric is likely robust enough to be used in future clinical trials. The semiquantitative image analysis of TI images had strong correlation with these measurements (Spearman’s rank correlation coefficient of 0.9). An important detail of both these analyses is that the graders were *not* from a professional reading center; rather, they were largely postundergraduate research assistants who were instructed in one session on the proper methodology. This process bodes well for reducing cost without sacrificing reliability in future studies.

The central hypothesis motivating this work is that an improvement in melanin synthesis in the RPE and/or choroid will positively affect the development of the human fovea in the adjacent neural retina during development. Some form of communication between these 2 tissue types must occur, as the genes that control melanin synthesis are expressed in pigmented cell types only and not in the neural retina itself. For the earliest steps of ocular development, such as ganglion cell differentiation, the developmental cues must come from the RPE, as the choroid has not yet formed and there is no melanin pigment in its presumptive location. An important distinction to make when stating this hypothesis is that it is likely not melanin per se that has the developmental effect, but, rather, the *process by which melanin in made*. As such, intermediates in the melanin synthesis pathway and/or indirect effects of melanin production may play a critical role.

A significant degree of work has focused on L-DOPA, an intermediate in melanin synthesis, as a potential RPE-derived mediator of neural retina development (28). Experiments in rodents suggest that there is a significant temporal lag in the differentiation/maturation in the retinas of albino animals. This delay in neurogenesis leads, among other findings, to an altered specification of the ventrotemporal retinal ganglion cells in albino mice; this altered specification is likely a major factor in the misrouting of the axons of these cells at the level of the optic chiasm (29, 30). Indeed, ectopic expression of tyrosine hydroxylase — which makes L-DOPA but not melanin pigment — in the RPE of albino mice can rescue the axonal crossing defect and improve their visual behavior (31). L-DOPA binds to GPR143 (the aberrant protein in X-linked albinism/*OA1*), possibly leading to the release of pigment epithelium-derived growth factor and/or other mediators of retinal development (32). The effects of L-DOPA on foveal maturation have not been well studied, as the common animal models used do not develop foveae. Finally, a randomized, double-masked, placebo-controlled trial of 2 doses of L-DOPA in patients with albinism has been completed (33). Although this trial did not show a statistically significant improvement in visual acuity over the 20 weeks of treatment, post hoc subgroup analysis suggested that younger children with worse visual acuity at study entry may have shown a trend towards improvement. In the present study, nitisinone would be predicted to indirectly increase local levels of L-DOPA by increasing flux through the melanin synthetic pathway. The mild improvement in visual acuity of our subjects is consistent with this hypothesis. As such, we similarly might observe clinically significant improvement in visual function with the treatment of children with nitisinone.

The trend of improved melanin synthesis in the hair and skin of OCA-1B patients treated with nitisinone is intriguing and warrants further study. Cutaneous damage from UV light puts patients with albinism, in particular, at an increased risk of skin cancers. This effect is perhaps most notable in areas of sub-Saharan Africa close to the equator, where UV exposure is high, a significant amount of time is spent out of doors, and founder mutations in *OCA2* result in a particularly high prevalence of albinism (34–38). We are encouraged that our data suggest an increase in eumelanin with a decrease in pheomelanin — precisely the effect desired to reduce skin cancer risk in OCA2 (12). Finally, improving skin and hair pigmentation in OCA2 in Africa could have important social implications, as persons with albinism are often stigmatized, abandoned, and maimed/murdered for their body parts, which are used for witchcraft purposes (35). For these reasons, our group is currently focused on testing nitisinone in preclinical animal models of this form of albinism.

## Methods

### Clinical trial design.

Five adult subjects with molecularly confirmed OCA-1B were enrolled in a 1-year, open-label pilot trial of oral nitisinone (Orfadin, NTBC) at the Clinical Center of the National Eye Institute of the NIH (NCT01838655, https://www.clinicaltrials.gov/) (Table 1), where all data were collected. Patients were recruited from an existing natural history cohort at the National Human Genome Research Institute (Clinical, Cellular and Molecular Investigations into Oculocutaneous Albinism [09-HG-0035]); additionally they were self-referred or referred from other ophthalmology practices.

OCA-1B was defined by the following: (a) bilateral visual acuity ETDRS–electronic visual acuity (ETDRS-EVA) letter score of ≤83 (i.e., Snellen equivalents of 20/25 or worse) that was not attributable to any other pathology (39); (b) bilateral iris TI that can be seen in clinical photographs; (c) predominant contralateral decussation of ganglion cell axons, as determined by pattern visual-evoked potential; (d) at least one definitive mutation in the *TYR* gene (tyrosinase); and (e) no definitive mutations in the *OCA2* gene. Formal inclusion and exclusion criteria are listed in Supplemental Table 1.

All participants received 2 mg oral nitisinone per day (self-administered), taken at least 1 hour before a meal or at least 2 hours after a meal. This dose is identical to that in the aforementioned trial in patients with alkaptonuria (23). They underwent a minimum of 8 appointments over a period of approximately 18 months (2 baseline visits, at months 3, 6, 9, 12, and 15 as well as a safety follow-up visit). All appointments, other than the 2 baseline visits, were conducted within a window of ±1 month from the target visit date. The 2 baseline visits were conducted on separate days, no more than 6 months apart. If necessary, the testing for each visit could be conducted on consecutive days. The following data were collected at study visits: (a) medical history and systemic physical examination; (b) ETDRS-EVA; (c) slit lamp examination and dilated fundus examination; (d) binocular contrast sensitivity under 3 glare conditions; (e) IOP; (f) color photography of the skin/hair pigmentation, iris pigmentation, iris TI, and the posterior pole; (g) skin reflectometry (40); (h) spectral domain optical coherence tomography; (i) blood work, including electrolyte and hepatic panels, complete blood count, serum plasma amino acids, nitisinone levels, and pregnancy test (for females); (j) hair sample collection, cut at base of the shaft; and (k) urinalysis. Participants kept pill diaries, which were reviewed at each visit, and met with a nutritionist for dietary guidance. No specific dietary restrictions were requested, other than avoiding diets that deliberately increased the amount of protein consumed (e.g., the “Atkins diet”). Female participants had a screening gynecologic exam and were counseled on effective means of birth control. Formal neuropsychological and neurology assessments were performed and visual functioning questionnaire (VFQ-39) was administered at baseline and at 12 months. Scotopic and photopic full-field ERG using standard ISCEV protocols were performed at baseline, 6 months, and 12 months.

### Iris pigmentation on an 8-point iris TI scale.

The primary outcome of this study was the absolute mean change in iris pigmentation on an 8-point scale at 12 months compared with baseline (24). Although other published scales of iris TI are useful in clinical practice (41), they do not appear to have the granularity required for a semiquantitative outcome variable in a clinical trial. A secondary analysis of absolute mean change at 3, 6, and 9 months compared with baseline was simultaneously performed using the same scale and methodology. High-resolution (3504 × 2336) digital images of the anterior segment of both eyes were captured prior to pupil dilation using (a) diffuse illumination and (b) iris TI. The Haag-Strait BX900 Photo Slit-Lamp and Canon EOS-20D camera running OIS WinStation 4000SLTM software were used. Iris TI photography was performed at ×10 with both the diffusion lens and the background illumination light off. The illumination arm was decoupled from the microscope, allowing the image beam to be centered on the pupil. Initially the beam was set to a 3-mm circle to fill the pupil, but it may have been reduced to a 2-mm or 1-mm circular beam if the participant’s pupil was smaller. The microscope aperture was set to 1 for optimal image exposure, and the flash was set on high. An independent reviewer selected 2 TI images of each eye of each participant for each visit according to preset quality criteria and saved them as uncompressed TIF files. Images were coded, randomized, and presented to a panel of 18 graders on a SHARP 90-inch HD LED TV. After instruction and a practice data set, graders scored each image using the 8-point scale (24). Graders could score images with a single decimal place if they felt an image fell in between 2 of the standards.

### EVA.

Visual acuity was assessed according to the ETDRS-EVA protocol. Manifest refraction was performed at baseline and 12 months, and if there was a ≥10 ETDRS-EVA letter (≥0.2 LogMAR) change from baseline.

### Electroretinography.

Full-field electroretinographies (ERGs) were recorded from bipolar Burian-Allen electrodes according to the standards specified by the International Society for Clinical Electrophysiology of Vision (ISCEV) (24). ERG amplitudes and implicit times of ERG components were measured and reported according to ISCEV standards. The ERG was recorded in this study as a measure of safety to assess whether retinal function was altered by nitisinone administration.

### Skin reflectometry.

Skin melanin concentrations were assessed using the M index, as outlined in Wagner et al. (40). Reflectance measurements were obtained at 10-nm increments along the visual spectrum from 400 to 700 nm using a HunterLab MiniScan EZ Model 4000S (Reston,Va), a diffuse reflectance spectrophotometer that uses a 256-element diode array, and a high-resolution, concave holographic grating. Taking measurements over a range of wavelengths allows for selection of wavelengths that are attributable to a skin characteristic of interest (here melanin content), as opposed to other potential causes for altered skin reflectance, e.g., erythema. Measurements were taken 5 times at each of the following body sites: forehead, inner forearm, outer forearm, inner bicep, and lower back. Non-sun-exposed locations such as the inner bicep are generally used to assess the degree of constitutive melanin content, as sun-exposed skin melanin likely reflects a combination of constitutive melanin and neomelanin (i.e., tanning). Before and after each set of measurements, the spectrophotometer was calibrated using reflectance from a standard white surface and a standard light trap (no reflectance). An estimate of the proportion of reflected light attributable to melanin, the M index, was then calculated as follows:

Eqn 1 = [(PR_650 nm_ + PR_60 nm_ + 0.5 × PR_640 nm_ + 0.5 × PR_670 nm_)/3]/100

M index = 100 × log_10_ (1/Eqn 1)

with PR representing proportion of reflected light.

Qualitative assessments of changes in hair, skin, and fundus pigmentation were performed by direct observation of the patient and comparison with photos (generally, front, back, and top of head) from previous visits.

### Hair melanin content.

PTCA, a marker of eumelanin, and 4-AHP, a marker of pheomelanin, were used to determine hair melanin content according to previously published HPLC protocols (42, 43). In brief, hair samples (11–13 mg) were homogenized in water with Tenbroeck homogenizer (Corning) at a concentration of 10 mg/ml, and 100-μl aliquots were subjected to alkaline hydrogen peroxide oxidation (42) and hydroiodic acid hydrolysis (43) before HPLC analysis.

### Measurement of nitisinone by LC-MS/MS.

Nitisinone was measured by liquid chromatography/tandem mass spectrometry (LC-MS/MS) (44, 45). A Waters Quattro Micro mass spectrometer coupled to an Agilent 1100 HPLC was used for analysis. 20 μl plasma sample was acidified with 0.2% formic acid (FA) in water and then treated with acidified acetonitrile (0.2% FA) containing mesotrione as the internal standard. The plasma proteins were precipitated and the samples were centrifuged. The supernatant was then injected into an LC-MS/MS system in positive electrospray ionization mode. Nitisinone and mesotrione were separated by an acetonitrile/water (0.1% FA) gradient using a Waters SymmetryShield RP18 column, 3.5-μm particle size, and 2.1 × 30 mm column size or a Waters Acquity UPLC BEH C18 1.7-μm, 2.1 × 30 mm column. Analytes were quantified by appropriate multiple reaction monitoring transitions: 320.1 > 218.0 for nitisinone, 340.1 > 228.0 for mesotrione IS.

### Semiquantitative iris TI image analysis.

Semiquantitative TI image analysis was performed using Adobe Photoshop CC (2015 edition). The marquee tool was used to draw a circle around the high-resolution TIF image of the transilluminated iris, and its center was marked by intersecting vertical and horizontal lines. Each quadrant of the iris was then further subdivided into 4 equal squares whose center was marked by a standard-sized circle (1.5 × 1.5 or 150 pixels/150 pixels; Supplemental Figure 2). A Gaussian blur (radius 50) was applied to each marked location, and the level of red signal was measured using the dropper tool. The average of these values was transformed to a scale of 0–8 and represents the iris TI score by this method.

### Statistics.

All statistical analyses were based on the 5 enrolled participants who completed 18 months of follow-up, including 12 months of nitisinone treatment, unless otherwise specified. Since this was a proof-of-principle study, all analyses were exploratory in nature.

Improvement or worsening in outcome measures were primarily assessed through descriptive statistics of change from baseline at follow-up visits. The earliest baseline visit with data available was used in the calculation of change from baseline.

Longitudinal analysis models were fit to assess whether a significant improvement or worsening was observed at the end of treatment (i.e., month 12) compared with baseline for select parameters. For this longitudinal analysis, the outcome was modeled starting at baseline through relevant follow-up visits (i.e., follow-up visits with sufficient data). Time (visit) was included as a fixed effect. Subject-specific (conditional) models were also fit; such models can distinguish observations belonging to the same or different participants. This was achieved by including the following random effects in the model: a random intercept that accounts for variation in baseline outcome values between participants and a random slope that accounts for variation in rate of change in outcome values during the course of the study between participants. Compound symmetry variance-covariance structure was assumed, i.e., variance in outcome measurements at each visit was assumed to be the same and correlation between measurements across visits was assumed to be the same, regardless of the time gap between visits. For models that consider values from both eyes of a participant together, eyes were nested within participants. Since all analyses were considered exploratory in this pilot study, adjustment for type I error was not performed. All *P* values reported are based on longitudinal analysis performed as described above. To aid interpretation, change at month 12 was considered significant if the *P* value from the statistical test was less than 0.05 and moderately significant if the *P* value was less than 0.10. Due to the limited sample size in this study, the results of longitudinal analyses cannot be considered confirmatory.

To informally assess the reliability of agreement among the 18 independent graders, ICC (46) with 95% CI was calculated at each visit by eye. The 18 graders were assumed to be a random sample of graders in the calculation of ICCs. ICCs were based on the mean of the grades assigned to the 2 images for each participant’s eye at each visit by each grader. The correlation of the grades assigned by the human graders with the grades assigned through semiquantitative iris TI image analysis was also assessed.

The National Eye Institute VFQ-39 subjective assessment of visual function was administered to subjects at each visit; VFQ-39 total and subscale scores were calculated as outlined by Mangione (47).

### Study approval.

This trial was approved by the Combined Neuroscience Institutional Review Board of the NIH and conducted in accordance with the Declaration of Helsinki. This project has been funded in part with federal funds from the National Eye Institute, NIH, Department of Health and Human Services, under contract HHSN263201700001C.

## Author contributions

DRA, PWC, SI, KW, RMJ, WJI, WAG, and BPB designed the trial and/or laboratory experiments. DRA, RJ, ZMV, BP, BGJ, AG, RPA, DC, EW, MAM, PWC, SB, SI, KW, RMJ, and BPB conducted the trial and/or laboratory experiments. DRA, SM, RJ, ZMV, BP, MA, EW, PWC, SI, KW, RMJ, and BPB analyzed the data. DRA, SM, RJ, MA, BGJ, DC, KW, RMJ, WAG, and BPB wrote the manuscript.

## Supplementary Material

Supplemental data

## Figures and Tables

**Table 5:**
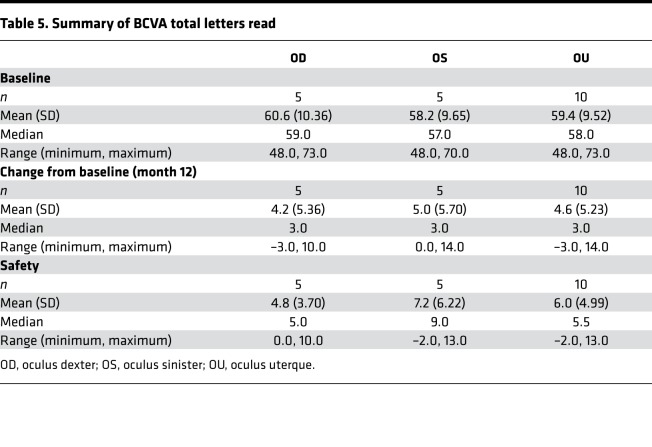
Summary of BCVA total letters read

**Table 4:**
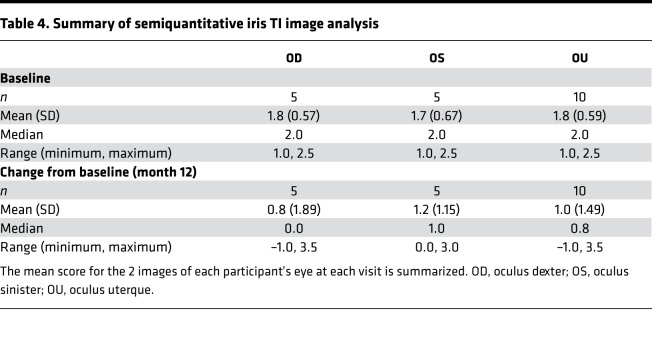
Summary of semiquantitative iris TI image analysis

**Table 3:**
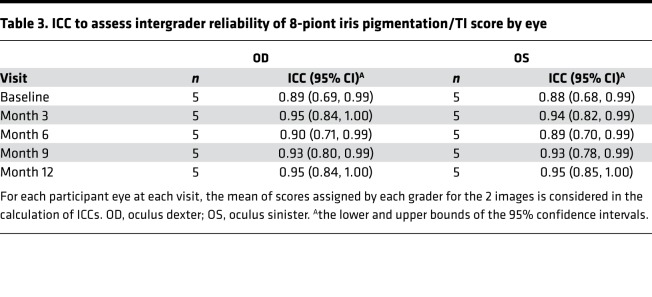
ICC to assess intergrader reliability of 8-piont iris pigmentation/TI score by eye

**Table 2:**
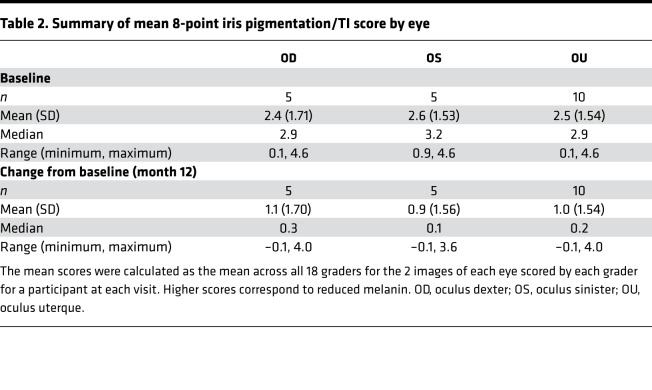
Summary of mean 8-point iris pigmentation/TI score by eye

**Table 1:**
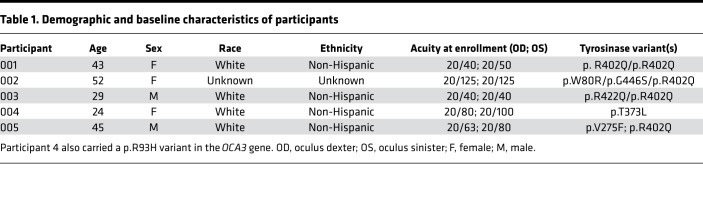
Demographic and baseline characteristics of participants

## Version 1 01/24/2019

Electronic publication

## Version 2 03/19/2019

Updated copyright information.
