# Supplementary material for: One-year pilot study on the effects of nitisinone on melanin in patients with OCA-1B
Source: JCI Insight. 2019 Jan 24;4(2):e124387. doi: 10.1172/jci.insight.124387 (PMC6413781; doi:10.1172/jci.insight.124387)
Supplement: Supplemental data [file jciinsight-4-124387-s012.pdf]

SUPPLEMENTARY TABLES

INCLUSION CRITERIA:
1. Age≥ 18 years
2. Ability to understand and sign consent
3. Normal renal and liver functions, platelet counts (no greater than grade 1 as defined by the Common Terminology Criteria for Adverse Events (CTCAE))
4. Negative pregnancy test (screening, enrollment, study visits) for females
5. Two effective methods of contraception while on NTBC
6. Signs and symptoms of OCA1b (defined in text)

EXCLUSION CRITERIA:
1. Pregnant or breast-feeding
2. Male AND a definitive mutation in *OA1* (*GPR143*)
3. Any of the following abnormal laboratory tests: serum potassium <3.0mEq/L; serum creatin kinase > 500U/L; hemoglobin < 10.0 g/dL; white blood cell count < 3.0 k/μL; plasma tyrosine > 150μM; ESR > 100mm/h; and/or serum T4 > 15μg/dL or <4μg/dL
4. Chronic keratopathy
5. Current malignancy
6. Open skin lesions
7. Diet that deliberately increases protein intake to disproportionate levels (e.g., Atkins diet).
8. Uncontrolled hypertension (systolic blood pressure > 180 mmHg or diastolic blood pressure > 95mm Hg)
9. Chronic ocular disease that may confound the results of visual tests (e.g., macular degeneration, cataract of possible visual significance, uncontrolled glaucoma).
10. Drinks > the equivalent of 2 glasses of wine/day or a history of alcohol abuse
11. Liver disease or live > 3cm below the right costal margin
12. Muscle disease
13. Medication known to cause elevated liver function (e.g., HMG Co-A reductase inhibitors, tetracycline, amiodarone, chronic acetaminophen)

Table S1: Inclusion and exclusion criteria

**System Organ Class**<br>**Preferred Term**	**Number of Events**<br>**N=10**<br>**N(%)**
**Participants enrolled**	5
**Participants with at least one adverse event**[a]	5 (100%)
Blood and lymphatic system disorders[b]	1 (10%)
Anaemia	1 (10%)

System Organ Class<br>Preferred Term	Number of Events<br>N=10<br>N(%)
Cardiac disorders[b]	1 (10%)
Palpitations	1 (10%)
Gastrointestinal disorders[b]	2 (20%)
Dyspepsia	1 (10%)
Gastrooesophageal reflux disease	1 (10%)
General disorders and administration site conditions[b]	1 (10%)
Chest pain	1 (10%)
Infections and infestations[b]	1 (10%)
Bronchitis	1 (10%)
Investigations[b]	1 (10%)
Neurological examination abnormal	1 (10%)
Musculoskeletal and connective tissue disorders[b]	1 (10%)
Pain in extremity	1 (10%)
Neoplasms benign, malignant and unspecified (incl cysts and polyps)[b]	1 (10%)
Haemangioma	1 (10%)
Nervous system disorders[b]	1 (10%)
Dizziness	1 (10%)

Table S2: Reported treatment-emergent adverse events. [a]Denominator is the number of participants enrolled. [b]Denominator is the number of events.

	Baseline	Change at Month 12
**Total Score[a]**		
N	5	5
Mean (SD)	73.7 (4.99)	5.1 (3.32)
Median	73.3	5.5
Range (Min, Max)	67.8, 80.5	0.6, 8.6
**General Health**		
N	5	5
Mean (SD)	82.0 (10.06)	3.0 (9.59)
Median	77.5	0.0
Range (Min, Max)	77.5, 100.0	-5.0, 17.5
**General Vision**		
N	5	5
Mean (SD)	54.0 (16.36)	15.0 (21.21)
Median	60.0	0.0
Range (Min, Max)	35.0, 75.0	0.0, 45.0
**Ocular Pain**		
N	5	5
Mean (SD)	82.5 (20.92)	15.0 (16.30)
Median	87.5	12.5
Range (Min, Max)	50.0, 100.0	0.0, 37.5
**Near Activities**		
N	5	5
Mean (SD)	70.8 (7.80)	4.2 (9.32)
Median	70.8	4.2
Range (Min, Max)	58.3, 79.2	-8.3, 16.7

	Baseline	Change at Month 12
**Distance Activities**		
N	5	5
Mean (SD)	59.2 (17.78)	2.0 (13.43)
Median	58.3	-6.8
Range (Min, Max)	41.7, 79.2	-8.3, 16.7
**Social Functioning**		
N	5	5
Mean (SD)	81.7 (16.03)	-1.7 (3.73)
Median	83.3	0.0
Range (Min, Max)	58.3, 100.0	-8.3, 0.0
**Mental Health**		
N	5	5
Mean (SD)	70.0 (5.00)	4.0 (6.52)
Median	70.0	0.0
Range (Min, Max)	65.0, 75.0	0.0, 15.0
**Role Difficulties**		
N	5	5
Mean (SD)	63.8 (14.25)	3.8 (19.06)
Median	62.5	0.0
Range (Min, Max)	50.0, 81.3	-18.8, 31.3
**Dependency**		
N	5	5
Mean (SD)	80.0 (8.15)	3.8 (11.35)
Median	81.3	6.3

	Baseline	Change at Month 12
Range (Min, Max)	68.8, 87.5	-12.5, 18.8
**Driving**		
N	5	5
Mean (SD)	83.3 (15.59)	-10.0 (16.03)
Median	83.3	-8.3
Range (Min, Max)	58.3, 100.0	-33.3, 8.3
**Color Vision**		
N	5	5
Mean (SD)	100.0 (0.00)	0.0 (0.00)
Median	100.0	0.0
Range (Min, Max)	100.0, 100.0	0.0, 0.0
**Peripheral Vision**		
N	5	5
Mean (SD)	65.0 (13.69)	20.0 (11.18)
Median	75.0	25.0
Range (Min, Max)	50.0, 75.0	0.0, 25.0

Table S3: Summary of VFQ-39 total and sub-scale scores.

SUPPLEMENTARY FIGURES:

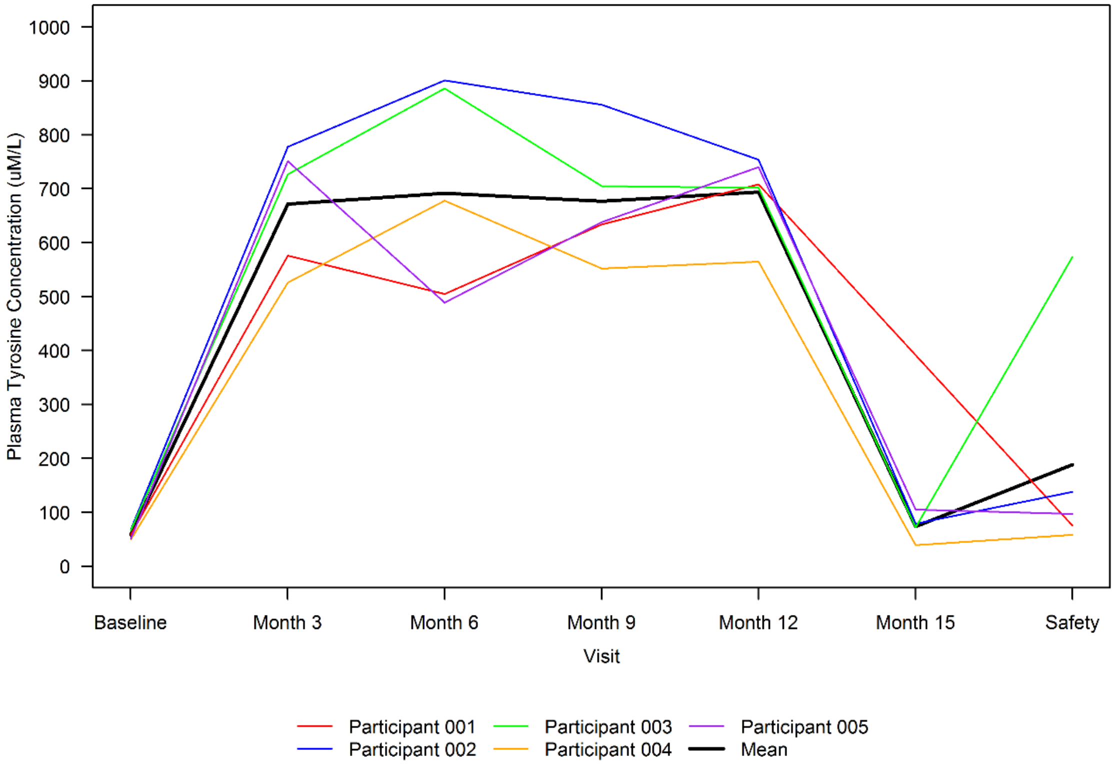

Figure S1: Plasma tyrosine concentration over time by participant and overall. The colored lines correspond to indivdiual participant measurements and the black line corresponds to mean (n=4 for Month 15; n=5 otherwise) across all participants. Plasma concentrations of tyrosine peaked between Months 3 and 12 for participants and dropped to near-baseline level at Month 15, which is three months after investigational product (IP) discontinuation. One participant chose the option to re-start IP at Month 18, before discontinuing it approximately 1.5 months later and completing their safety follow-up visit, resulting in the spike in mean plasma nitisinone concentration at the safety follow-up visit.

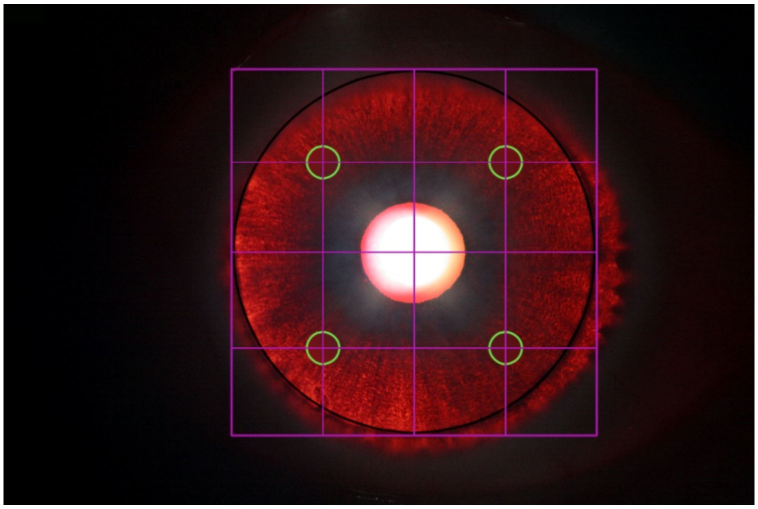

Figure S2: Diagram of the method of semiquantitative measurement of iris transillumination. The red signal from the circled areas were averaged to find a transillumination value for each image.
